# The Expression and Effection of MicroRNA-499a in High-Tobacco Exposed Head and Neck Squamous Cell Carcinoma: A Bioinformatic Analysis

**DOI:** 10.3389/fonc.2019.00678

**Published:** 2019-07-31

**Authors:** Shi-Qi Gong, Meng Xu, Ming-Liang Xiang, Ya-Min Shan, Hao Zhang

**Affiliations:** ^1^Department of Otolaryngology, Ruijin Hospital, School of Medicine, Shanghai Jiaotong University, Shanghai, China; ^2^Department of Radiation Oncology, The First Affiliated Hospital, Anhui Medical University, Hefei, China

**Keywords:** lifetime tobacco exposure value, head and neck squamous cell carcinoma, microRNA-499a, bioinformatic analysis, WGCNA

## Abstract

**Background:** Few studies have directly investigated the differential expression of microRNAs (miRNAs) in head and neck squamous cell carcinoma (HNSCC) with low, medium, and high tobacco exposure. The purpose of this study is to screen the differentially expressed miRNAs and to investigate their clinical significance and potential biological mechanisms in the three groups of HNSCC.

**Methods:** The datasets of HNSCC were obtained from The Cancer Genome Atlas (TCGA). The edgeR package was used to determine differentially expressed miRNAs and genes among the three groups of HNSCC. Statistical methods were applied to assess the clinical significance of miRNA and its correlation with genes. The correlation between gene expression and clinical characteristics was analyzed using weighted gene co-expression network analysis (WGCNA). Three online databases were used to predict the target genes of miRNAs. More importantly, qRT-PCR was employed to verify the differential expression of miRNAs and genes in our patients.

**Results:** 32 differentially expressed miRNAs and 1,820 differentially expressed genes were found among the three groups of HNSCC. Patients with high expression of hsa-miR-499a had lower overall survival than the ones with low expression in high-tobacco exposed HNSCC. Cox regression analysis found that high expression of hsa-miR-499a and female were independent risk factors for prognosis in high-tobacco exposed HNSCC. Chi-square test found that hsa-miR-499a was associated with N stage in high-tobacco exposed HNSCC. WGCNA identified four gene modules associated with N stage in high-tobacco exposed HNSCC. Then three online databases were used to predict potential target genes for hsa-miR-499a, which were AEBP2 and ZNRF1. Pearson correlation analysis showed that hsa-miR-499a was negatively correlated with AEBP2 and ZNRF1. qRT-PCR supported bioinformatic results that hsa-miR-499a, AEBP2, and ZNRF1 were differentially expressed among the three groups of HNSCC in our patients.

**Conclusion:** 32 differentially expressed miRNAs and 1,820 differentially expressed genes were successfully identified in HNSCC with low, medium, and high tobacco exposure. The patients with high expression of hsa-miR-499a had poor prognoses compared with patients with low expression in high-tobacco exposed HNSCC. Hsa-miR-499a was associated with N stage in high-tobacco exposed HNSCC. AEBP2 and ZNRF1 were the potential target genes of hsa-miR-499a.

## Introduction

Head and neck squamous cell carcinoma (HNSCC) is the sixth most common human cancer (1). Most patients have locally advanced disease, and more than 50% of patients relapse within 3 years (2). About two-thirds of HNSCC patients have advanced disease, usually involving regional lymph nodes metastasis (3). HNSCC remains a clinical challenge, especially for patients diagnosed with the advanced or recurrent disease, HNSCC is almost fatal (4). Although significant efforts have been made to understand HNSCC, its mechanisms of occurrence and development has not yet been elucidated. Obviously, the exact mechanisms for exploring the occurrence and development of HNSCC is of great significance.

On one hand, to date, many studies have shown that tobacco exposure is an important risk factor for HNSCC (5–8). Cumulative tobacco tar exposure value is closely related to HNSCC (9). On the other hand, abnormal expression of miRNA has now been demonstrated in many cancer types (10, 11). The correlation between the abnormal expression of some miRNAs and outcome of HNSCC patients is indeed reported (12, 13). However, the role of miRNAs in HNSCC patients with different level of tobacco exposure remains unknown.

To further explore the relationship between patient clinical characteristics and gene expression, we apply co-expression network analysis. Weighted gene co-expression network analysis (WGCNA) is widely used to analyze large-scale datasets and to find modules for highly related genes (14), and it is successfully used to explore the association between gene expression and clinical characteristics, and to identify candidate biomarkers (15).

The present study combined and analyzed the miRNA-seq data, gene expression data (mRNA-seq data) and clinical data in The Cancer Genome Atlas (TCGA) database. Using bioinformatic analysis, this study identified differentially expressed miRNAs and genes in HNSCC with low, medium, and high tobacco exposure, and explored the possible role of hsa-miR-499a in high-tobacco exposed HNSCC. WGCNA was used to predict potential gene modules associated with N stage of high-tobacco exposed HNSCC. The potential target genes of hsa-miR-499a, i.e., AEBP2, ZNRF1, were predicted using three online databases and Pearson correlation analysis. In addition, we observed the independent effect of lifetime tobacco exposure value on the expression of hsa-miR-499a, AEBP2, and ZNRF1, excluding the effect of age at initial diagnosis. Furthermore, we validated the differential expression of hsa-miR-499a, AEBP2, and ZNRF1 in HNSCC patients with low, medium, and high tobacco exposure using tumor tissues of HNSCC patients from our research center by qRT-PCR.

## Materials and Methods

### Patient Selection, miRNA and Gene Screening

The HNSCC datasets were downloaded from TCGA (https://cancergenome.nih.gov/). Our research included laryngeal, hypopharyngeal, oropharyngeal, and tonsillar carcinomas of HNSCC. A total of 182 HNSCC patients were found in TCGA database, which included 118 patients with lifetime tobacco exposure value. Those HNSCC patients with complete information containing miRNA-seq data, gene expression data (mRNA-seq data) and clinical data could be included in our study. Ten HNSCC patients were excluded due to lack of miRNA-seq or mRNA-seq data. And finally, 108 HNSCC patients were selected for subsequent analysis, and their clinical characteristics were shown in Supplementary Table 1.

In TCGA database, numerically calculated values represent lifetime tobacco exposure, defined as the number of cigarettes per day multiplied by the number of years of smoking divided by 20. The HNSCC patients were categorized into three groups based on the lifetime tobacco exposure value which was <30 for low-tobacco exposed HNSCC (*n* = 31), ≥30 and <50 for medium-tobacco exposed HNSCC (*n* = 36), and ≥50 for high-tobacco exposed HNSCC (*n* = 41).

With normalized data, analysis of differentially expressed miRNAs and genes was performed using the edgeR package in R software. The fold change (FC), *P*-value and false discovery rate (FDR) were calculated. Next, the differentially expressed miRNAs we found in the three groups of HNSCC were further divided into low and high expressions with the median of each miRNA expression level as the cut-off value in low-tobacco, medium-tobacco, and high-tobacco exposed HNSCC, respectively (Supplementary Table 2).

To verify the bioinformatic analysis results, we further collected 22 cases of smoking patients with HNSCC (including 15 cases of laryngeal cancer, 4 cases of hypopharyngeal cancer, 1 case of oropharyngeal cancer, and 2 cases of tonsillar cancer) from the Ruijin Hospital, School of Medicine, Shanghai Jiaotong University. Our HNSCC patients (or their parents or guardians) have signed the written informed consent form. The use of human tissue samples and clinical data has been approved by the Ruijin Hospital Ethics Committee. Similarly, we used the lifetime tobacco exposure value to group patients. The patients with lifetime tobacco exposure value <30 were included in low-tobacco exposed group, ≥30 and <50 were included in medium-tobacco exposed group, and ≥50 were included in high-tobacco exposed group (including 4 low-tobacco exposed HNSCC, 6 medium-tobacco exposed HNSCC, and 12 high-tobacco exposed HNSCC). We collected patient's tumor specimens and recorded the patient's clinical characteristics. The general characteristics of these patients were comparable (Supplementary Table 3). The entire study design followed appropriate medical ethics principles.

### Association of miRNAs With Patients' Prognosis and Clinical Characteristics

Kaplan-Meier survival analysis (log-rank method) and univariate/multivariate Cox proportional hazard regression analysis were used to explore the relationship between each parameter and overall survival of the patients in TCGA database. Chi-square test was used to assess the relationship between differentially expressed miRNAs and clinical characteristics of patients.

### Gene Expression Profiling Data Processing

Gene expression data (mRNA-seq data) was obtained from TCGA database. A total of 32,216 genes were identified for each sample. The variance analysis was performed, and it was ranked from large to small. The top 25% of the genes (8,054 genes) with larger variance were selected for WGCNA analysis.

### WGCNA Co-expression Network Construction

The expression data profile of these 8,054 genes was constructed to a gene co-expression network using the WGCNA package in R software (15). An adjacency matrix was constructed using the WGCNA function adjacency by calculating the Pearson correlation between all pairs of genes in all selected samples. In this study, the power of β = 7 (scale-free R^2^ = 0.9) was used as a soft threshold parameter to ensure a scale-free network. To further identify functional modules in the co-expression network with these 8,054 genes, the adjacency matrix was used to calculate the topological overlap measurement (TOM) representing the overlap in the shared neighbors.

### Identification of Clinical Significant Modules

The module eigengenes (MEs) were considered to be a representation of the gene expression profile in the module. Correlation and *P*-value between the module and clinical characteristics were evaluated by calculating the MEs. In the correlation between the module and clinical characteristics, red represents a positive correlation with clinical characteristics, and green represents a negative correlation with clinical characteristics.

### Gene Ontology and Pathway Enrichment Analysis

The Database for Annotation, Visualization and Integrated Discovery (DAVID) is a database for annotating gene functions and visualizing them. Gene ontology (GO) and Kyoto Encyclopedia of Genes and Genomes (KEGG) pathway analysis of the genes within significant modules were performed using the DAVID (16) (version 6.8; https://david.ncifcrf.gov/) online tool: Functional Annotation Tool. The ontology contained three categories: biological processes (BP), cellular components (CC), and molecular functions (MF). Enriched GO terms and KEGG pathways were determined based on the adjusted critical criteria of *P* < 0.05.

### Predicting the Target Gene of miRNA

The potential target genes of the miRNAs were predicted using TargetScan (17) (http://www.targetscan.org/vert_72/), miRDB (18) (http://www.mirdb.org/), and DIANA (19) (diana.imis.athena-innovation.gr/DianaTools/index.php) online analysis tools. To further enhance the reliability of bioinformatic analysis, we performed Pearson correlation analysis between miRNAs and genes. Venn diagrams were used to identify overlapping target genes.

### Differential Expression Analysis Among Three Different Age Groups of Non-smoking HNSCC

Because there were differences in age at initial diagnosis among the low-tobacco, medium-tobacco, and high-tobacco exposed HNSCC (Supplementary Table 1, *P* = 0.02), we performed this additional analysis. Sixty four non-smoking HNSCC patients were found in TCGA database. Those HNSCC patients with complete information containing miRNA-seq data, gene expression data (mRNA-seq data) and clinical data could be included in this additional analysis. Five non-smoking patients were excluded due to lack of miRNA-seq or mRNA-seq data. And finally, 59 non-smoking HNSCC patients were selected for this additional analysis, and their clinical characteristics were shown in Supplementary Table 1.

The non-smoking patients were categorized into three groups based on the age at initial diagnosis which was ≤ 55 for low-age group (*n* = 19), >55 and <65 for medium-age group (*n* = 21), ≥65 for high-age group (*n* = 19). With normalized data, analysis of differentially expressed miRNAs and genes was performed using the edgeR package in R software.

### Quantitative Reverse Transcription-Polymerase Chain Reaction (qRT-PCR)

Total RNA was isolated from HNSCC samples using TRIzol reagent. MiRNA first strand cDNA synthesis (Tailing Reaction) and microRNAs qPCR (SYBR Green Method) kits were purchased from Sangon Biotech for miRNA qRT-PCR reactions. HiScript III RT SuperMix for qPCR Kit and ChamQ Universal SYBR qPCR Master Mix were purchased from Vazyme Biotech for mRNA qRT-PCR reactions. MiRNA levels were normalized to U6 gene expression, mRNA levels were normalized to GAPDH gene expression, and relative expression levels were analyzed using the ΔCt method. The primers were designed and purchased from Sangon Biotech. The following primers were used: hsa-miR-499a-5p, forward, 5′-GCGCGTTAAGACTTGCAGTGATGTTT-3′; hsa-miR-499a-3p, forward, 5′-CGAACATCACAGCAAGTCTGTGCT-3′; AEBP2, forward, 5′-CAGCAGTAGCAGCAGCGTAGTC-3′ and reverse, 5′-CCATCCGACGACATCTCCAAGC-3′; ZNRF1, forward, 5′-ACGATGATGTGCTGACTAAAGA-3′ and reverse, 5′-TTCACTTCAAACCACGAGTCTA-3′.

### Statistical Analysis

Statistical analysis was performed using SPSS Statistics software (version 24.0) and R software (version 3.4.2; https://www.r-project.org/). *P*-value <0.05 was considered statistically significant. The column diagram was graphed with Graphpad Prism software (version 7.0a).

## Results

### Differentially Expressed miRNAs and Genes

From TCGA database, we noted that 1,881 miRNAs and 32,216 genes were analyzed for each patient. After restricting the cut-off criteria (logFC > 1, FDR < 0.05), we found 3 differentially expressed miRNAs and 637 differentially expressed genes between low-tobacco and medium-tobacco exposed HNSCC, 23 differentially expressed miRNAs and 805 differentially expressed genes between medium-tobacco and high-tobacco exposed HNSCC, and 25 differentially expressed miRNAs and 1,038 differentially expressed genes between low-tobacco and high-tobacco exposed HNSCC (Tables 1–3 and Supplementary Table 4). These results contain 32 differentially expressed miRNAs and 1,820 differentially expressed genes.

**Table 1:** Differentially expressed miRNAs (Medium-tobacco exposed HNSCC compared with low-tobacco exposed HNSCC).

**miRNAs**	**logFC**	**logCPM**	***P*-value**	**FDR**
hsa-mir-1224	3.7296	1.3577	**7.94E-08**	**4.96E-05**
hsa-mir-5683	3.4463	3.7835	**2.14E-06**	**0.0006**
hsa-mir-3923	−4.7849	0.9820	**2.83E-06**	**0.0006**
hsa-mir-499a	1.1367	1.4493	**0.0124**	0.3034

*FC, fold change; CPM, count-per-million; FDR, false discovery rate. The P-value indicating statistical significance is marked with bold type*.

**Table 2:** Differentially expressed miRNAs (High-tobacco exposed HNSCC compared with medium-tobacco exposed HNSCC).

**miRNAs**	**logFC**	**logCPM**	***P*-value**	**FDR**
hsa-mir-508	3.2473	8.0556	**4.14E-06**	**0.0026**
hsa-mir-592	2.1092	3.1726	**1.40E-05**	**0.0045**
hsa-mir-509-2	2.9787	5.0636	**2.50E-05**	**0.0053**
hsa-mir-552	3.4295	0.4420	**3.45E-05**	**0.0055**
hsa-mir-503	−1.3728	3.4885	**7.08E-05**	**0.0075**
hsa-mir-499a	2.7289	1.8473	**7.83E-05**	**0.0075**
hsa-mir-4728	−1.6964	0.9378	**8.27E-05**	**0.0075**
hsa-mir-509-1	2.9353	5.0400	**9.72E-05**	**0.0077**
hsa-mir-466	2.1942	0.0030	**0.0002**	**0.0111**
hsa-mir-514a-2	2.8532	5.4999	**0.0002**	**0.0119**
hsa-mir-506	3.3164	2.5576	**0.0002**	**0.0119**
hsa-mir-514a-1	2.7431	5.4671	**0.0002**	**0.0125**
hsa-mir-490	−3.5387	−0.0705	**0.0003**	**0.0132**
hsa-mir-514a-3	2.7054	5.4875	**0.0003**	**0.0132**
hsa-mir-509-3	2.6975	5.2508	**0.0003**	**0.0132**
hsa-mir-129-1	1.7561	3.5999	**0.0003**	**0.0132**
hsa-mir-219a-2	3.1774	−0.0234	**0.0004**	**0.0132**
hsa-mir-20b	−1.5453	5.4539	**0.0006**	**0.0191**
hsa-mir-129-2	1.7021	3.6655	**0.0011**	**0.0312**
hsa-mir-507	2.6516	−0.1118	**0.0015**	**0.0370**
hsa-mir-6715a	−2.6129	0.0576	**0.0015**	**0.0370**
hsa-mir-513c	2.3608	0.5594	**0.0019**	**0.0419**
hsa-mir-514b	2.4332	0.5602	**0.0020**	**0.0419**

*FC, fold change; CPM, count-per-million; FDR, false discovery rate. The P-value indicating statistical significance is marked with bold type*.

**Table 3:** Differentially expressed miRNAs (High-tobacco exposed HNSCC compared with low-tobacco exposed HNSCC).

**miRNAs**	**logFC**	**logCPM**	***P*-value**	**FDR**
hsa-mir-499a	3.9279	1.6513	**7.60E-07**	**0.0005**
hsa-mir-508	3.8674	8.0973	**2.04E-06**	**0.0006**
hsa-mir-3923	−4.3025	0.8479	**3.28E-06**	**0.0007**
hsa-mir-129-1	2.4443	3.5526	**4.73E-06**	**0.0007**
hsa-mir-129-2	2.4922	3.6003	**6.36E-06**	**0.0007**
hsa-mir-509-2	3.6996	5.0794	**7.60E-06**	**0.0007**
hsa-mir-509-3	3.9127	5.2276	**7.87E-06**	**0.0007**
hsa-mir-1224	2.3775	0.4683	**1.36E-05**	**0.0011**
hsa-mir-20b	−1.8981	5.6071	**1.79E-05**	**0.0013**
hsa-mir-506	4.4612	2.5809	**2.55E-05**	**0.0016**
hsa-mir-509-1	3.7382	5.0492	**2.79E-05**	**0.0016**
hsa-mir-204	2.9815	4.6620	**5.45E-05**	**0.0029**
hsa-mir-507	3.6491	−0.1031	**0.0001**	**0.0052**
hsa-mir-552	3.4765	0.5348	**0.0001**	**0.0052**
hsa-mir-573	−2.0547	0.3892	**0.0001**	**0.0052**
hsa-mir-514a-2	3.1784	5.5570	**0.0002**	**0.0069**
hsa-mir-5683	2.5020	3.0818	**0.0002**	**0.0069**
hsa-mir-143	1.0878	16.4992	**0.0002**	**0.0069**
hsa-mir-514a-3	3.2830	5.5200	**0.0002**	**0.0069**
hsa-mir-514a-1	3.1591	5.5147	**0.0002**	**0.0069**
hsa-mir-363	−1.5342	4.7707	**0.0004**	**0.0115**
hsa-mir-592	1.7889	3.3192	**0.0009**	**0.0213**
hsa-mir-4521	−1.2676	0.7194	**0.0013**	**0.0278**
hsa-mir-885	1.7879	0.1884	**0.0016**	**0.0319**
hsa-mir-219a-2	2.5408	0.0825	**0.0022**	**0.0413**

*FC, fold change; CPM, count-per-million; FDR, false discovery rate. The P-value indicating statistical significance is marked with bold type*.

Hsa-miR-499a was upregulated in high-tobacco exposed group compared to low-tobacco (logFC = 3.9279, FDR = 0.0005) and medium-tobacco (logFC = 2.7289, FDR = 0.0075) exposed groups. Although hsa-miR-499a had a trend to upregulate in medium-tobacco exposed group compared to low-tobacco exposed group (logFC = 1.1367, *P* = 0.0124), we could not currently consider hsa-miR-499a to be upregulated in medium-tobacco exposed group due to FDR = 0.3034. Meanwhile, we found that AEBP2 was downregulated in medium-tobacco (logFC = −1.4748, FDR = 2.53E-07) and high-tobacco (logFC = −3.8258, FDR = 1.40E-07) exposed groups compared to low-tobacco exposed group, ZNRF1 was downregulated in high-tobacco exposed group compared to low-tobacco (logFC = −2.6838, FDR = 0.0011) and medium-tobacco (logFC = −1.8838, FDR = 0.0003) exposed groups.

### Prognostic Value of Differentially Expressed miRNAs in the Three Groups of HNSCC

**Figure 1:**
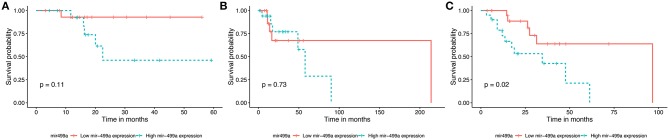
Survival curve for the three groups of HNSCC using Kaplan-Meier analyses (log-rank method). In low-tobacco (**A**, *P* = 0.11) and medium-tobacco (**B**, *P* = 0.73) exposed HNSCC, the patients with various expression levels of hsa-miR-499a were not different in overall survival. In high-tobacco exposed HNSCC, the patients with low expression of hsa-miR-499a had higher overall survival rates than the ones with high expression (**C**, *P* = 0.02).

The survival data were extracted from TCGA database. We identified the effects of 32 differentially expressed miRNAs on survival rate in the three groups of HNSCC. We found that patients with low expression of hsa-miR-499a (Figure 1) and hsa-miR-129-2 (Supplementary Figure 1), and high expression of hsa-miR-508 (Supplementary Figure 2) had higher survival rates than the ones with high expression of hsa-miR-499a and hsa-miR-129-2, and low expression of hsa-miR-508 in high-tobacco exposed HNSCC. While different expression levels of hsa-miR-499a, hsa-miR-129-2 and hsa-miR-508 had no effect on patients' survival in low-tobacco and medium-tobacco exposed HNSCC. Twenty nine other miRNAs were not found to have an effect on overall survival in the three groups of HNSCC (*P* > 0.05).

In addition, we evaluated the prognostic value of hsa-miR-499a, hsa-miR-129-2, hsa-miR-508, and the clinical parameters in the three groups of HNSCC patients by univariate and multivariate Cox proportional hazard regression analysis. We found that high expression of hsa-miR-499a (HR, 3.28) and female (HR, 3.49) were risk factors for prognosis in high-tobacco exposed HNSCC. Furthermore, high expression of hsa-miR-499a (HR, 3.26) and female (HR, 3.38) were found to be independent risk factors for prognosis in high-tobacco exposed HNSCC (Table 4). We did not find the prognostic value of hsa-miR-499a in low-tobacco and medium-tobacco exposed HNSCC (Supplementary Table 5). However, we did not find the prognostic value of hsa-miR-129-2, hsa-miR-508 in the three groups of HNSCC (*P* > 0.05). Therefore, we chose hsa-miR-499a for further analysis.

**Table 4:** Univariate and multivariate Cox proportional hazard regression analysis for high-tobacco exposed HNSCC.

**Variables**	**Univariate analysis**		**Multivariate analysis**	
	**HR(95%CI)**	***P*-value**	**HR(95%CI)**	***P*-value**
Age at initial diagnosis (≥60)	0.51(0.16,1.68)	0.268		
Gender (Female)	3.49(1.29,9.45)	**0.014**	3.38(1.25,9.15)	**0.017**
Histologic grade (G3/G1+G2)	0.34(0.07,1.58)	0.168		
Pathologic stage (IV/I+II+III)	0.43(0.12,1.53)	0.195		
T stage (T3+T4/T1+T2)	0.40(0.12,1.28)	0.122		
N stage (N1+N2+N3/N0)	4.06(0.89,18.62)	0.071		
M stage (M1+Mx/M0)	1.39(0.33,5.92)	0.653		
hsa-mir-499a (High expression)	3.28(1.13,9.50)	**0.029**	3.26(1.11,9,59)	**0.032**
hsa-mir-129-2 (High expression)	3.07(0.96,9.84)	0.059	2.86(0.86,9.50)	0.086
hsa-mir-508 (High expression)	0.36(0.13,1.01)	0.053	0.38(0.13,1.12)	0.079

*HR, hazard ratio. The P-value indicating statistical significance is marked with bold type*.

### Identifying the Clinical Significance of hsa-miR-499a in the Three Groups of HNSCC

To identify the clinical significance of hsa-miR-499a, we evaluated the association between the expression of hsa-miR-499a and clinical characteristics of the patients with high-tobacco exposure (Table 5), low-tobacco and medium-tobacco exposure (Supplementary Table 6). In high-tobacco exposed HNSCC, the expression of hsa-miR-499a was associated with N stage (*P* < 0.01). In low-tobacco and medium-tobacco exposed HNSCC, no correlation between the expression of hsa-miR-499a and clinical characteristics were found.

**Table 5:** The correlations between hsa-mir-499a and the characteristics of high-tobacco exposed HNSCC.

**Variables**	**High-tobacco exposed HNSCC**		
	**Total (%)**	**Low/High expression level**	***P*-value**
**AGE AT INITIAL DIAGNOSIS**			
<60	11(26.83%)	5/6	0.66
≥60	30(73.17%)	16/14	
**GENDER**			
Male	31(75.61%)	17/14	0.65
Female	10(24.39%)	4/6	
**HISTOLOGIC GRADE**			
G1+G2	33(80.49%)	17/16	1.00
G3	8(19.51%)	4/4	
Gx	0(0%)	0/0	
**PATHOLOGIC STAGE**			
I+II+III	6(14.63%)	2/4	0.39
IV	25(60.98%)	14/11	
NA	10(24.39%)	5/5	
**T STAGE**			
T1+T2	6(14.63%)	1/5	0.17
T3+T4	26(63.41%)	15/11	
Tx	7(17.07%)	4/3	
NA	2(4.88%)	1/1	
**N STAGE**			
N0	11(26.83%)	9/2	** <0.01**
N1-3	20(48.78%)	6/14	
Nx	8(19.51%)	5/3	
NA	2(4.88%)	1/1	
**M STAGE**			
M0	15(36.59%)	8/7	0.58
M1	1(2.44%)	0/1	
Mx	3(7.32%)	1/2	
NA	22(53.66%)	12/10	

*NA, Not Applicable. The P-value indicating statistical significance is marked with bold type*.

### Weighted Co-expression Network Construction and Key Modules Identification

**Figure 2:**
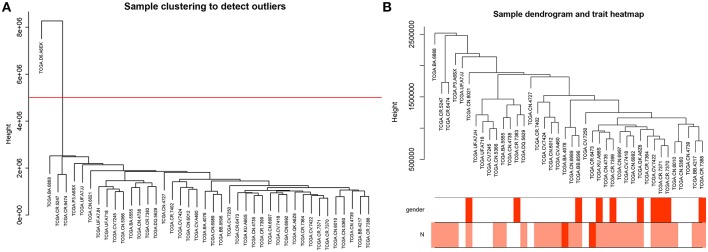
**(A)** Clustering dendrogram of 41 samples, and excluding 1 outlier sample. **(B)** Clustering dendrogram of 40 samples corresponding to clinical characteristics.

To further explore the genes associated with N stage in high-tobacco exposed HNSCC, we performed a WGCNA analysis. The samples of high-tobacco exposed HNSCC patients (*n* = 41) were clustered using average linkage method and Pearson correlation method. We excluded 1 outlier sample and finally included 40 samples for analysis (Figure 2).

**Figure 3:**
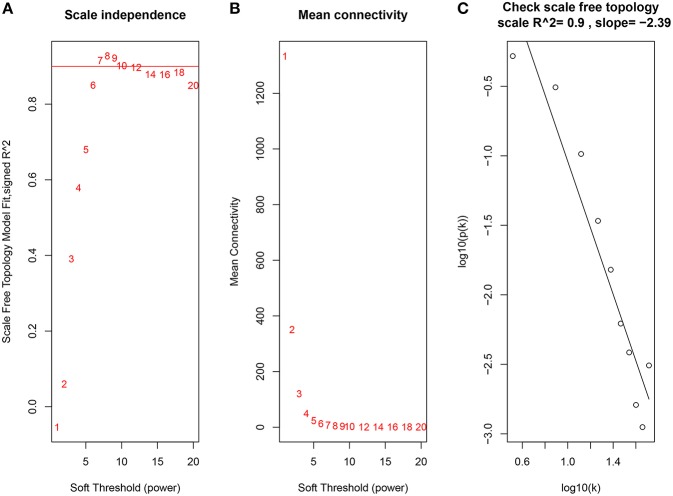
Determination of soft-thresholding power in the WGCNA. **(A)** Analysis of the scale-free fit index for various soft-thresholding powers (β). **(B)** Analysis of the mean connectivity for various soft-thresholding powers. **(C)** Checking the scale free topology when β = 7.

Constructing a WGCNA needs the best soft-thresholding power to which co-expression similarity is raised to calculate adjacency. Therefore, we performed a network topology analysis of various soft-thresholding powers to have relatively balanced scale independence and average connectivity of WGCNA. In this study, the power of β = 7 (scale-free R^2^ = 0.9) was selected as the soft-thresholding parameter to ensure a scale-free network (Figure 3).

**Figure 4:**
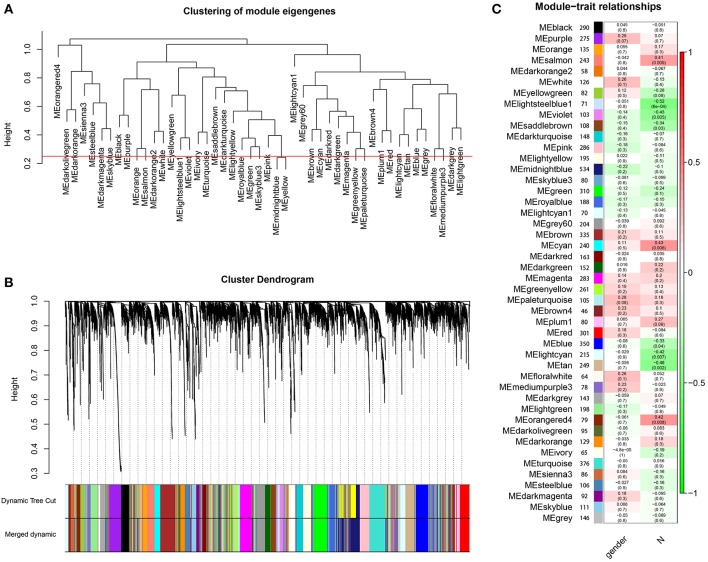
Identification of modules associated with the clinical characteristics of high-tobacco exposed HNSCC. **(A)** The horizontal line defined the threshold, so 46 distinct gene modules were identified. **(B)** Dendrogram of all genes were clustered based on a dissimilarity measure (1-TOM). **(C)** Heatmap of the correlation between module eigengenes and clinical characteristics of high-tobacco exposed HNSCC.

Through dynamic tree cut and merged dynamics, 47 different gene modules were generated in a hierarchical clustering tree from 40 samples, and each module marked by a different color was displayed through a tree diagram, wherein each tree branch constituted a module and each leaf in the branch was one gene. As shown in Figure 4A, the horizontal line defined the threshold, by merging similar modules, 46 distinct gene modules were identified (Figure 4B). We selected the modules that were negatively correlated with N stage and *P* < 0.01. Four modules (lightsteelblue1, tan, violet, lightcyan) were found to have association with N stage (Figure 4C, Supplementary Table 7), and those modules were selected as the significant modules for further analysis.

### GO and KEGG Pathway Enrichment Analysis

**Figure 5:**
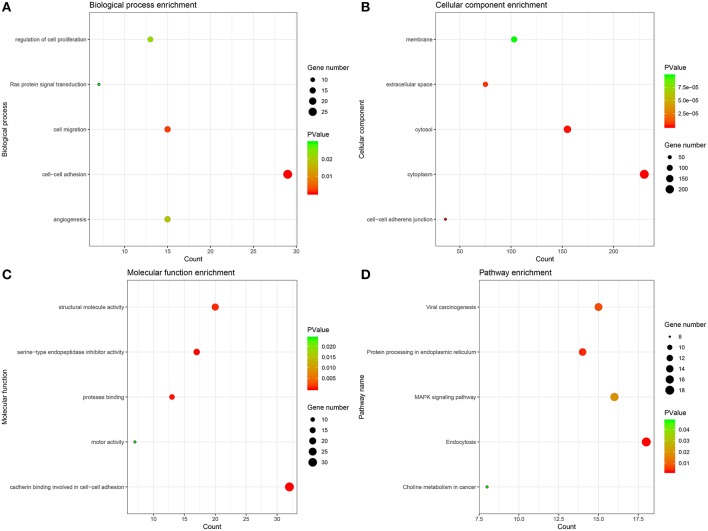
GO and KEGG pathway enrichment analysis. **(A)** Biological process analysis. **(B)** Cellular component analysis. **(C)** Molecular function analysis. **(D)** KEGG pathway analysis.

The genes of significant modules were categorized into 3 functional groups, i.e., BP, CC, and MF. The genes in the BP group were mainly enriched in cell-cell adhesion, cell migration, angiogenesis, regulation of cell proliferation, and Ras protein signal transduction. The genes in the CC group were significantly enriched in cell-cell adherens junction, cytoplasm, cytosol, extracellular space, and membrane. The genes in the MF group were mainly enriched in cadherin binding involved in cell-cell adhesion, serine-type endopeptidase inhibitor activity, protease binding, structural molecule activity and motor activity. According to KEGG pathway analysis, our results demonstrated that these genes were mainly involved in MAPK signaling pathway, protein processing in endoplasmic reticulum, viral carcinogenesis, endocytosis and choline metabolism in cancer. These results indicated that the genes of significant modules were mainly involved in cell-cell adherens junction (Figure 5, Supplementary Table 8).

### Predicting Potential Target Genes of hsa-miR-499a

**Figure 6:**
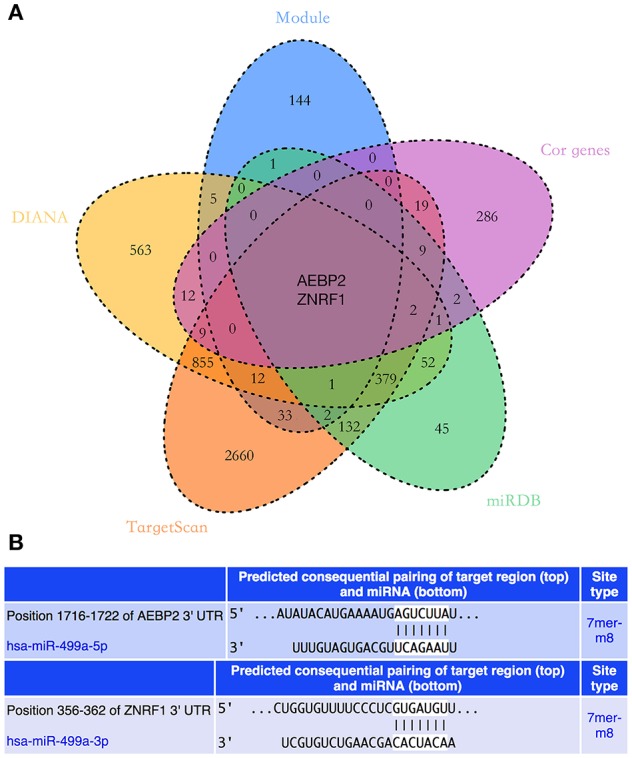
**(A)** The Venn diagram showed the intersection of three online databases, four significant modules, and genes which were correlated with hsa-miR-499a. **(B)** The predicted binding sites of hsa-miR-499a to the 3′UTR region of AEBP2 and ZNRF1.

The potential target genes of hsa-miR-499a were predicted using three online databases we introduced above. Next, we selected the top 50 genes in each significant module according to the degree of connectivity within the module. To improve the accuracy of the prediction, we performed the Pearson correlation analysis between hsa-miR-499a and 1,820 genes differentially expressed among the three groups of HNSCC. According to *P* < 0.05, we found 342 genes associated with hsa-miR-499a, in which AEBP2 (Cor = −0.343, *P* = 0.028) and ZNRF1 (Cor = −0.330, *P* = 0.035) were negatively correlated with hsa-miR-499a (Supplementary Table 9). Finally, we took the intersection of the above three results. AEBP2 and ZNRF1 were identified as potential target genes for hsa-miR-499a (Figure 6A).

### Independent Effect of Lifetime Tobacco Exposure Value on the Expression of hsa-miR-499a, AEBP2, and ZNRF1

There were differences in age at initial diagnosis among the low-tobacco, medium-tobacco, and high-tobacco exposed HNSCC (Supplementary Table 1, *P* = 0.02). In order to further determine the independent effect of lifetime tobacco exposure value on the expression of hsa-miR-499a, AEBP2, and ZNRF1, we performed differential expression analysis among the three non-smoking groups of HNSCC. After excluding the effect of tobacco exposure, we did not find differential expression of hsa-miR-499a, AEBP2, and ZNRF1 between low-age group and medium-age group, medium-age group and high-age group, as well as low-age group and high-age group (Supplementary Table 10, FDR > 0.05). These results indicated that the differential expression of hsa-miR-499a, AEBP2, and ZNRF1 in HNSCC with low, medium, and high tobacco exposure was due to the different lifetime tobacco exposure value, rather than the different age at initial diagnosis.

### Validation of the Differential Expression of hsa-miR-499a, AEBP2, and ZNRF1

**Figure 7:**
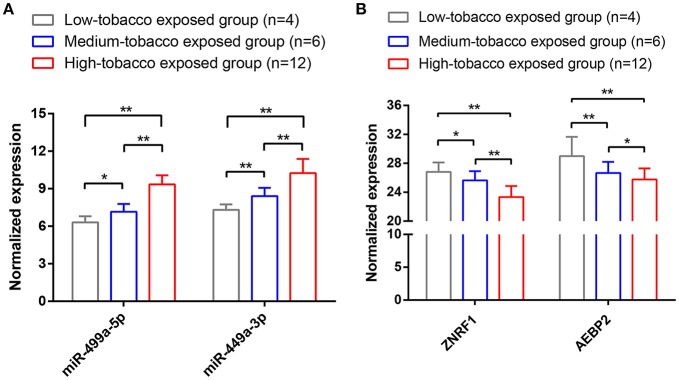
The results of qRT-PCR showed that **(A)** the expression of hsa-miR-499a-5p and hsa-miR-499a-3p in high-tobacco exposed HNSCC was higher than those in low-tobacco and medium-tobacco exposed HNSCC, and the expression of hsa-miR-499a-5p and hsa-miR-499a-3p in medium-tobacco exposed HNSCC was higher than those in low-tobacco exposed HNSCC. **(B)** The expression of AEBP2 and ZNRF1 in high-tobacco exposed HNSCC was lower than those in low-tobacco and medium-tobacco exposed HNSCC, and the expression of AEBP2 and ZNRF1 in medium-tobacco exposed HNSCC was lower than those in low-tobacco exposed HNSCC (^*^P < 0.05, ^**^P < 0.01).

To validate the results of bioinformatic analysis, we conducted a qRT-PCR for evaluation of hsa-miR-499a-5p, hsa-miR-499a-3p, AEBP2, and ZNEF1 expression levels of tumor samples in low-tobacco, medium-tobacco, and high-tobacco exposed HNSCC patients in our hospital. We found that the expression of hsa-miR-499a-5p and hsa-miR-499a-3p in high-tobacco exposed HNSCC was higher than those in low-tobacco and medium-tobacco exposed HNSCC, and the expression of hsa-miR-499a-5p and hsa-miR-499a-3p in medium-tobacco exposed HNSCC was higher than those in low-tobacco exposed HNSCC. Meanwhile, the expression of AEBP2 and ZNRF1 in high-tobacco exposed HNSCC was lower than those in low-tobacco and medium-tobacco exposed HNSCC, and the expression of AEBP2 and ZNRF1 in medium-tobacco exposed HNSCC was lower than those in low-tobacco exposed HNSCC (Figure 7). The qRT-PCR results validated the differentially expressed hsa-miR-499a, AEBP2, and ZNRF1 we found in the analysis of the TCGA datasets.

**Figure 8:**
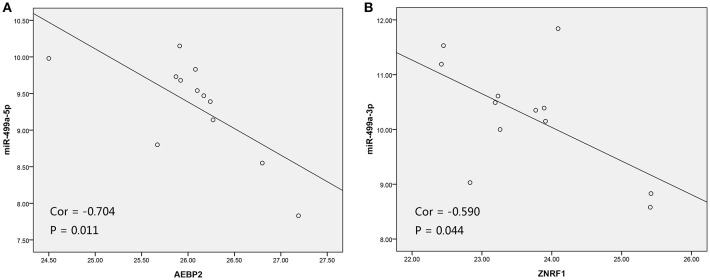
The scatter plot showed a negative correlation between hsa-miR-499a-5p and AEBP2 **(A)**, and the scatter plot showed a negative correlation between hsa-miR-499a-3p and ZNRF1 **(B)** in 12 high-tobacco exposed HNSCC patients of our hospital.

In addition, we used the TargetScan database to predict the binding site of hsa-miR-499a-5p to AEBP2, and the binding site of hsa-miR-499a-3p to ZNRF1 (Figure 6B). Then, we performed the Pearson correlation analysis of expression levels between hsa-miR-499a and AEBP2, ZNRF1 in our own patients. The results showed that hsa-miR-499a-5p was negatively correlated with AEBP2, and hsa-miR-499a-3p was negatively correlated with ZNRF1 (Figure 8, Table 6), which was consistent with the results from the analysis of TCGA datasets.

**Table 6:** The Pearson correlation analysis of expression levels between hsa-miR-499a and AEBP2, ZNRF1 in our own patients.

**miRNA**	**miR-499a-5p**		**miR-499a-3p**	
**Gene**	**AEBP2**	**ZNRF1**	**AEBP2**	**ZNRF1**
Cor	−0.704	0.153	−0.310	−0.590
*P*-value	**0.011**	0.636	0.327	**0.044**

*Cor, correlation coefficient. The P-value indicating statistical significance is marked with bold type*.

## Discussion

HNSCC is a serious hazard to human health, and it is easy to relapse even after comprehensive treatment. The 5-year overall survival rate of HNSCC patients is <50%. Although the treatment of HNSCC has improved over the past few decades, the prognosis of patients with advanced HNSCC remains poor. Therefore, it is extremely important to study the mechanisms of HNSCC occurrence and progression.

Previous studies have confirmed that smoking is an independent risk factor for the occurrence and progression of HNSCC (5–8), and lifetime cumulative tobacco tar exposure is associated with cancer risk (9). Most studies categorize tobacco exposure as “forever” and “never” or according to the number of years of smoking. The former does not consider the important impact of smoking cessation on the risk of tobacco-attributable cancer. The latter did not take into account the impact of smoking volume on the risk of cancer (20). In TCGA database, numerically calculated values represent lifetime tobacco exposure, defined as the number of cigarettes per day multiplied by the number of years of smoking divided by 20 (21, 22). Lifetime tobacco exposure value includes the number of cigarettes smoked and the time of smoking. Even patients who have quit smoking can still assess the effects of tobacco on tumorigenesis and tumor development. On the other hand, the abnormal expressions of miRNAs play an important role in HNSCC (23, 24). Studies have shown that the recruitment of miRNA-induced silencing complexes (miRISC) is linked to mRNA targets to reduce target protein production (25). However, in HNSCC with different tobacco exposure, the roles of miRNAs remain unknown. To our knowledge, this study is the first to use miRNA-seq data, gene expression data (mRNA-seq data) and clinical data from HNSCC patients in TCGA database to analyze differentially expressed miRNAs and genes in HNSCC with low, medium and high tobacco exposure, and to explore the relationship between differentially expressed miRNAs and clinical characteristics, and to predict the potential target genes of miRNA by WGCNA, online databases and Pearson correlation analysis.

After analyzing the TCGA datasets, we found 32 differentially expressed miRNAs and 1,820 differentially expressed genes among the three groups of HNSCC, including hsa-miR-499a, which was upregulated in high-tobacco exposed group compared to low-tobacco (logFC = 3.9279, FDR = 0.0005) and medium-tobacco (logFC = 2.7289, FDR = 0.0075) exposed groups. Although hsa-miR-499a had a trend to upregulate in medium-tobacco exposed group compared to low-tobacco exposed group (logFC = 1.1367, *P* = 0.0124), we could not currently consider hsa-miR-499a to be upregulated in medium-tobacco exposed group due to FDR = 0.3034 in the analysis of TCGA datasets. However, the qRT-PCR of our own HNSCC patients showed that the expression of hsa-miR-499a-5p and hsa-miR-499a-3p in high-tobacco exposed HNSCC was higher than those in low-tobacco and medium-tobacco exposed HNSCC, and the expression of hsa-miR-499a-5p and hsa-miR-499a-3p in medium-tobacco exposed HNSCC was higher than those in low-tobacco exposed HNSCC. Therefore, hsa-miR-499a has a potential tendency to increase expression with increasing tobacco exposure.

The results of differential expression analysis mean different tobacco exposed HNSCC may lead to different regulatory modes of miRNAs and genes. Most importantly, our research has gained new information that can guide further research. Previous other studies have shown that the 32 differentially expressed miRNAs we find are related to the biological characteristics of tumors. Such as hsa-miR-509 promotes cisplatin-induced apoptosis in ovarian cancer cells (26), hsa-miR-506 enhances the sensitivity of human colorectal cancer cells to oxaliplatin (27), hsa-miR-129 regulates cisplatin-resistance in human gastric cancer cells (28), hsa-miR-503 enhances the radiosensitivity of laryngeal carcinoma cells (29) and so on. These miRNAs are closely related to chemoradiotherapy sensitivity. Because of different tobacco exposure, miRNAs in HNSCC patients are differentially expressed, which means the clinical characteristics of patients may also be different, and the patient's treatment may change, for example, the way of chemoradiotherapy.

In our research, KM survival analysis and Cox regression showed that patients with high expression of hsa-miR-499a had a poor prognosis compared with the ones with low expression of hsa-miR-499a in high-tobacco exposed HNSCC. Chi-square test showed that hsa-miR-499a was associated with N stage in high-tobacco exposed HNSCC. In low-tobacco and medium-tobacco exposed HNSCC, we did not find the clinical significance of hsa-miR-499a.

It has been reported that hsa-miR-499a can have dual and opposite functions in various cancer types, either as a tumor suppressor miRNA or as a tumorigenic miRNA. Hsa-miR-499a is highly expressed in colorectal cancer patients with lymph node metastasis and promotes the migration and invasion of colorectal cancer by regulating the expression of FOXO4 and PDCD4 (30). In contrast, hsa-miR-499a inhibits the expression of EST1 and blocks the invasion and migration of HepG2 cells. This means that hsa-miR-499a may have tumor suppressor function in the pathogenesis of hepatocellular carcinoma by targeting ETS1 (31). ETS1 has been reported as an oncogene in previous studies, and in most cancers, ETS1 expression is associated with poor survival (32). Similarly, the expression of hsa-miR-499a is low in oral squamous cell carcinoma tissue compared with the corresponding adjacent normal tissue. It was also found that the expression level of hsa-miR-499a is related to advanced disease and survival rate (33). However, the role of hsa-miR-499a needs further investigation in HNSCC.

We further performed WGCNA in high-tobacco exposed HNSCC to explore gene co-expression modules associated with N stage. A total of 8,054 genes were used to construct a co-expression network and 46 modules were identified. We selected the modules that were negatively correlated with N stage and *P* < 0.01. Four modules (lightsteelblue1, tan, violet, lightcyan) were found to have association with N stage.

We used three online databases (DIANA, TargetScan, miRDB) to predict potential target genes of hsa-miR-499a. To improve the accuracy of the prediction, we performed the Pearson correlation analysis between hsa-miR-499a and 1,820 genes differentially expressed among the three groups of HNSCC. According to *P* < 0.05, we found 342 genes associated with hsa-miR-499a, in which AEBP2 (Cor = −0.343, *P* = 0.028) and ZNRF1 (Cor = −0.330, *P* = 0.035) were negatively correlated with hsa-miR-499a. After taking the intersection of the genes associated with N stage obtained by WGCNA, the target genes of hsa-miR-499a predicted by three online databases, and genes co-expressed with hsa-miR-499a, AEBP2 and ZNRF1 were found to be potential target genes for hsa-miR-499a. Some studies have reported the role of ZNRF1. CBX8 triggers the progression of hepatocellular carcinoma by increasing the expression of hsa-miR-365a-3p down-regulated ZNRF1 (34). Deletion of ZNRF1 may be involved in the mechanism of B-cell acute lymphoblastic leukemia associated with PAX5 alteration (35). This indicates that ZNRF1 is a tumor suppressor gene.

Studies have reported the function of AEBP2 in tumors. Deletion of other members of AEBP2 and polycomb suppression complex 2 is associated with secondary AML following chronic myeloproliferative neoplasms and myelodysplastic syndromes (36). The absence of AEBP2 is also pathologically important in children with new-onset AML (37). These results indicate that the absence of AEBP2 leads to the occurrence and progression of leukemia and AEBP2 is a tumor suppressor gene.

Furthermore, although there were differences in age at initial diagnosis among the low-tobacco, medium-tobacco, and high-tobacco exposed HNSCC, the additional analysis indicated that the differential expression of hsa-miR-499a, AEBP2, and ZNRF1 in HNSCC with low, medium, and high tobacco exposure was due to the different lifetime tobacco exposure value, rather than the different age at initial diagnosis.

In our bioinformatic analysis, hsa-miR-499a, AEBP2, and ZNRF1 were differentially expressed in HNSCC with low, medium, and high tobacco exposure. Our own patients' results also supported the differential expression of hsa-miR-499a, AEBP2, and ZNRF1 among the three groups of HNSCC by qRT-PCR.

Our current study is a preliminary investigation of the clinical significance and potential biological mechanisms of hsa-miR-499a in HNSCC with low, medium, and high tobacco exposure. We will further use our clinical samples to verify the clinical and prognostic value of hsa-miR-499a in HNSCC with low, medium, and high tobacco exposure. Our research may reveal the underlying mechanisms of hsa-miR-499a in HNSCC oncogenesis and provide novel strategy in HNSCC treatment.

## Conclusion

Our study successfully identified 32 differentially expressed miRNAs and 1,820 differentially expressed genes in HNSCC with low, medium, and high tobacco exposure. The patients with high expression of hsa-miR-499a had poor prognoses compared with patients with low expression in high-tobacco exposed HNSCC. Hsa-miR-499a was associated with N stage in high-tobacco exposed HNSCC. The potential target genes of hsa-miR-499a were identified as AEBP2 and ZNRF1 based on WGCNA, three online databases and Pearson correlation analysis. Even so, further clinical and experimental research are needed to verify these conclusions.

## Data Availability

The datasets generated for this study can be found in TCGA, https://cancergenome.nih.gov/

## Ethics Statement

Our HNSCC patients (or their parents or guardians) have signed the written informed consent form. The use of human tissue samples and clinical data has been approved by the Ruijin Hospital Ethics Committee.

## Author Contributions

S-QG is responsible for research design, data collection, bioinformatic analysis, qRT-PCR experiments, and manuscript writing. MX is responsible for data collection and statistical analysis. M-LX is responsible for providing tumor samples and guiding research ideas. Y-MS guides research ideas, data interpretation, and experimental methods. HZ guides research ideas, design, research methods, and manuscript revision.

### Conflict of Interest Statement

The authors declare that the research was conducted in the absence of any commercial or financial relationships that could be construed as a potential conflict of interest.
