# Supplementary material for: The Expression and Effection of MicroRNA-499a in High-Tobacco Exposed Head and Neck Squamous Cell Carcinoma: A Bioinformatic Analysis
Source: Front Oncol. 2019 Jul 31;9:678. doi: 10.3389/fonc.2019.00678 (PMC6685408; doi:10.3389/fonc.2019.00678)
Supplement: Supplementary file 1 [file Table_1.docx]

**Supplementary Table 1.** The characteristics of 108 smoking patients with HNSCC in TCGA database.

| Variables | Low-tobacco group | Medium-tobacco group | High-tobacco group | P value |
| --- | --- | --- | --- | --- |
| Age at initial diagnosis | |  |  |  |
| <60 | 18 | 18 | 11 | **0.02** |
| >=60 | 13 | 18 | 30 |  |
| Gender |  |  |  |  |
| Male | 28 | 30 | 31 | 0.26 |
| Female | 3 | 6 | 10 |  |
| Histologic grade |  |  |  |  |
| G1+G2 | 20 | 20 | 33 | 0.16 |
| G3 | 10 | 13 | 8 |  |
| Gx | 1 | 3 | 0 |  |
| Pathologic stage |  |  |  |  |
| I+II+III | 7 | 9 | 6 | 0.43 |
| IV | 19 | 17 | 25 |  |
| NA | 5 | 10 | 10 |  |
| T stage |  |  |  |  |
| T1+T2 | 10 | 9 | 9 | 0.68 |
| T3+T4 | 16 | 21 | 23 |  |
| Tx | 4 | 5 | 7 |  |
| NA | 1 | 1 | 2 |  |
| N stage |  |  |  |  |
| N0 | 12 | 10 | 11 | 0.76 |
| N1-3 | 15 | 17 | 20 |  |
| Nx | 2 | 8 | 8 |  |
| NA | 2 | 1 | 2 |  |
| M stage |  |  |  |  |
| M0 | 18 | 12 | 15 | 0.69 |
| M1+Mx | 6 | 6 | 4 |  |
| NA | 7 | 18 | 22 |  |
| Tumor type |  |  |  |  |
| Larynx | 22 | 26 | 26 | 0.98 |
| Hypopharynx | 2 | 2 | 3 |  |
| Oropharynx | 1 | 2 | 3 |  |
| Tonsil | 6 | 6 | 9 |  |

NA= Not Applicable; The P value indicating statistical significance is marked with bold type.

**Supplementary Table 1.** The characteristics of 59 non-smoking patients with HNSCC in TCGA database.

| Variables | Low-age group | Medium-age group | High-age group | P value |
| --- | --- | --- | --- | --- |
| Gender |  |  |  |  |
| Male | 15 | 17 | 17 | 0.65 |
| Female | 4 | 4 | 2 |  |
| Histologic grade |  |  |  |  |
| G1+G2 | 10 | 12 | 10 | 0.81 |
| G3 | 5 | 8 | 8 |  |
| Gx | 3 | 1 | 1 |  |
| NA | 1 | 0 | 0 |  |
| Pathologic stage |  |  |  |  |
| I+II+III | 4 | 7 | 6 | 0.88 |
| IV | 9 | 12 | 9 |  |
| NA | 6 | 2 | 4 |  |
| T stage |  |  |  |  |
| T1+T2 | 5 | 7 | 6 | 0.99 |
| T3+T4 | 9 | 12 | 10 |  |
| Tx | 2 | 1 | 1 |  |
| NA | 3 | 1 | 2 |  |
| N stage |  |  |  |  |
| N0 | 6 | 7 | 4 | 0.87 |
| N1-3 | 7 | 12 | 6 |  |
| Nx | 3 | 1 | 7 |  |
| NA | 3 | 1 | 2 |  |
| M stage |  |  |  |  |
| M0 | 7 | 6 | 5 | 0.77 |
| M1+Mx | 3 | 3 | 1 |  |
| NA | 9 | 12 | 13 |  |
| Tumor type |  |  |  |  |
| Larynx | 14 | 13 | 10 | 0.87 |
| Hypopharynx | 0 | 1 | 1 |  |
| Oropharynx | 1 | 1 | 1 |  |
| Tonsil | 4 | 6 | 7 |  |

NA= Not Applicable.
