# Supplementary material for: The Expression and Effection of MicroRNA-499a in High-Tobacco Exposed Head and Neck Squamous Cell Carcinoma: A Bioinformatic Analysis
Source: Front Oncol. 2019 Jul 31;9:678. doi: 10.3389/fonc.2019.00678 (PMC6685408; doi:10.3389/fonc.2019.00678)
Supplement: Supplementary file 2 [file Table_2.DOCX]

**Supplementary Table 2.** The cut-off values of 32 differentially expressed miRNAs.

| miRNA | Low-tobacco group | Medium-tobacco group | High-tobacco group |
| --- | --- | --- | --- |
| hsa-mir-499a | 0.5421 | 1.1173 | 2.4909 |
| hsa-mir-1224 | 0.6699 | 1.1767 | 1.2272 |
| hsa-mir-129-1 | 5.4498 | 6.0985 | 10.5941 |
| hsa-mir-129-2 | 3.9483 | 5.5105 | 12.1004 |
| hsa-mir-143 | 148638.0005 | 162431.5736 | 303721.7537 |
| hsa-mir-204 | 2.5393 | 7.6589 | 7.2948 |
| hsa-mir-20b | 98.5216 | 60.9106 | 29.4290 |
| hsa-mir-219a-2 | 0.0351 | 0.0065 | 0.0379 |
| hsa-mir-363 | 44.4613 | 40.2402 | 22.5248 |
| hsa-mir-3923 | 0.0298 | 0.0297 | 0.0222 |
| hsa-mir-4521 | 2.9747 | 2.9428 | 1.8973 |
| hsa-mir-466 | 0.0581 | 0.0385 | 0.2265 |
| hsa-mir-4728 | 2.5440 | 2.9461 | 1.7994 |
| hsa-mir-490 | 0.0240 | 0.016 | 0.0033 |
| hsa-mir-503 | 23.0052 | 21.6809 | 16.3188 |
| hsa-mir-506 | 0.0418 | 0.1832 | 0.2564 |
| hsa-mir-507 | 0.0061 | 0.0061 | 0.0346 |
| hsa-mir-508 | 16.1679 | 28.9622 | 19.8087 |
| hsa-mir-509-1 | 1.9954 | 3.5406 | 2.8292 |
| hsa-mir-509-2 | 2.6520 | 6.4336 | 3.3969 |
| hsa-mir-509-3 | 1.4413 | 3.6901 | 3.6023 |
| hsa-mir-513c | 0.0279 | 0.0309 | 0.1602 |
| hsa-mir-514a-1 | 1.9723 | 4.1557 | 2.7891 |
| hsa-mir-514a-2 | 2.1767 | 4.4863 | 3.6641 |
| hsa-mir-514a-3 | 1.9818 | 5.2048 | 3.5776 |
| hsa-mir-514b | 0.0214 | 0.0339 | 0.0797 |
| hsa-mir-552 | 0.0090 | 0.0116 | 0.0725 |
| hsa-mir-5683 | 2.6537 | 3.307 | 2.6952 |
| hsa-mir-573 | 1.2034 | 1.4076 | 0.1758 |
| hsa-mir-592 | 9.3538 | 7.6915 | 8.5500 |
| hsa-mir-6715a | 0.0228 | 0.0269 | 0.0163 |
| hsa-mir-885 | 0.5320 | 0.5653 | 0.7793 |
