# Supplementary material for: The Expression and Effection of MicroRNA-499a in High-Tobacco Exposed Head and Neck Squamous Cell Carcinoma: A Bioinformatic Analysis
Source: Front Oncol. 2019 Jul 31;9:678. doi: 10.3389/fonc.2019.00678 (PMC6685408; doi:10.3389/fonc.2019.00678)
Supplement: Supplementary file 3 [file Table_3.DOCX]

**Supplementary Table 3**. The characteristics of 22 HNSCC patients in our hospital.

| Variables | Low-tobacco group | Medium-tobacco group | High-tobacco group | P value |
| --- | --- | --- | --- | --- |
| Age at initial diagnosis | |  |  |  |
| <60 | 3 | 3 | 3 | 0.18 |
| >=60 | 1 | 3 | 9 |  |
| Gender |  |  |  |  |
| Male | 4 | 5 | 11 | 0.66 |
| Female | 0 | 1 | 1 |  |
| Histologic grade |  |  |  |  |
| G1+G2 | 3 | 4 | 5 | 0.40 |
| G3 | 1 | 2 | 7 |  |
| Pathologic stage |  |  |  |  |
| I+II+III | 3 | 2 | 7 | 0.40 |
| IV | 1 | 4 | 5 |  |
| T stage |  |  |  |  |
| T1+T2 | 2 | 4 | 6 | 0.78 |
| T3+T4 | 2 | 2 | 6 |  |
| N stage |  |  |  |  |
| N0 | 3 | 4 | 7 | 0.82 |
| N1-3 | 1 | 2 | 5 |  |
| M stage |  |  |  |  |
| M0 | 4 | 6 | 12 | \ |
| M1 | 0 | 0 | 0 |  |
| Tumor type |  |  |  |  |
| Larynx | 2 | 4 | 9 | 0.28 |
| Hypopharynx | 2 | 1 | 1 |  |
| Oropharynx | 0 | 1 | 0 |  |
| Tonsil | 0 | 0 | 2 |  |
