# Supplementary material for: The Expression and Effection of MicroRNA-499a in High-Tobacco Exposed Head and Neck Squamous Cell Carcinoma: A Bioinformatic Analysis
Source: Front Oncol. 2019 Jul 31;9:678. doi: 10.3389/fonc.2019.00678 (PMC6685408; doi:10.3389/fonc.2019.00678)
Supplement: Supplementary file 5 [file Table_5.docx]

**Supplementary Table 5.** Univariate and multivariate Cox proportional hazard regression analysis for low-tobacco exposed HNSCC.

| Variables | Univariate analysis | | Multivariate analysis | |
| --- | --- | --- | --- | --- |
|  | HR(95%CI) | P value | HR(95%CI) | P value |
| Age at initial diagnosis (>=60) | 0.63(0.07,5.45) | 0.674 |  |  |
| Gender (Female) | 0.04(0.01,6.54) | 0.615 |  |  |
| Histologic grade (G3/G1+G2) | 2.56(0.43,15.39) | 0.304 |  |  |
| Pathologic stage (IV/I+II+III) | 1.85(0.21,16.65) | 0.584 |  |  |
| T stage (T3+T4/T1+T2) | 1.52(0.25,9.15) | 0.648 |  |  |
| N stage (N1+N2+N3/N0) | 5.39(0.43,10.15) | 0.442 |  |  |
| M stage (M1+Mx/M0) | 3.13(0.19,13.97) | 0.422 |  |  |
| hsa-mir-499a (High expression) | 4.85(0.56,12.70) | 0.351 |  |  |
| hsa-mir-129-2 (High expression) | 1.48(0.09,2.66) | 0.403 |  |  |
| hsa-mir-508 (High expression) | 0.41(0.08,2.23) | 0.301 |  |  |

HR = hazard ratio.

**Supplementary Table 5.** Univariate and multivariate Cox proportional hazard regression analysis for medium-tobacco exposed HNSCC.

| Variables | Univariate analysis | | Multivariate analysis | |
| --- | --- | --- | --- | --- |
|  | HR(95%CI) | P value | HR(95%CI) | P value |
| Age at initial diagnosis (>=60) | 0.61(0.16,2.29) | 0.465 |  |  |
| Gender (Female) | 2.27(0.56,9.24) | 0.252 |  |  |
| Histologic grade (G3/G1+G2) | 0.85(0.20,3.65) | 0.823 |  |  |
| Pathologic stage (IV/I+II+III) | 4.07(0.04,9.71) | 0.301 |  |  |
| T stage (T3+T4/T1+T2) | 2.35(0.44,12.41) | 0.316 |  |  |
| N stage (N1+N2+N3/N0) | 1.21(0.29,5.47) | 0.804 |  |  |
| M stage (M1+Mx/M0) | 1.19(0.11,11.24) | 0.885 |  |  |
| hsa-mir-499a (High expression) | 1.25(0.35,4.46) | 0.730 |  |  |
| hsa-mir-129-2 (High expression) | 1.32(0.38,4.57) | 0.666 |  |  |
| hsa-mir-508 (High expression) | 0.80(0.23,2.76) | 0.719 |  |  |

HR = hazard ratio.
