# Supplementary material for: The Expression and Effection of MicroRNA-499a in High-Tobacco Exposed Head and Neck Squamous Cell Carcinoma: A Bioinformatic Analysis
Source: Front Oncol. 2019 Jul 31;9:678. doi: 10.3389/fonc.2019.00678 (PMC6685408; doi:10.3389/fonc.2019.00678)
Supplement: Supplementary file 6 [file Table_6.docx]

**Supplementary Table 6.** The correlations between hsa-mir-499a and the clinical characteristics in low-tobacco and medium-tobacco exposed HNSCC.

| Variables | Low-tobacco exposed HNSCC | | | Variables | Medium-tobacco exposed HNSCC | | |
| --- | --- | --- | --- | --- | --- | --- | --- |
|  | Total (%) | Low/High expression level | P value |  | Total (%) | Low/High expression level | P value |
| Age at initial diagnosis | | |  | Age at initial diagnosis | | |  |
| <60 | 24 | 11/13 | 0.39 | <60 | 12 | 6/6 | 1.00 |
| >=60 | 7 | 5/2 |  | >=60 | 24 | 12/12 |  |
| Gender |  |  |  | Gender |  |  |  |
| Male | 28 | 14/14 | 1.00 | Male | 30 | 18/12 | 0.37 |
| Female | 3 | 1/2 |  | Female | 6 | 2/4 |  |
| Histologic grade | |  |  | Histologic grade | |  |  |
| G1+G2 | 20 | 12/8 | 0.44 | G1+G2 | 20 | 10/10 | 0.72 |
| G3 | 10 | 4/6 |  | G3 | 13 | 5/8 |  |
| Gx | 1 | 0/1 |  | Gx | 3 | 3/0 |  |
| Pathologic stage | |  |  | Pathologic stage | |  |  |
| I+II+III | 7 | 5/2 | 0.66 | I+II+III | 9 | 4/5 | 1.00 |
| IV | 19 | 10/9 |  | IV | 17 | 9/8 |  |
| NA | 5 | 1/4 |  | NA | 10 | 5/5 |  |
| T stage |  |  |  | T stage |  |  |  |
| T1+T2 | 13 | 7/6 | 1.00 | T1+T2 | 9 | 3/6 | 0.42 |
| T3+T4 | 16 | 9/7 |  | T3+T4 | 18 | 10/8 |  |
| Tx | 1 | 0/1 |  | Tx | 8 | 5/3 |  |
| NA | 1 | 0/1 |  | NA | 1 | 0/1 |  |
| N stage |  |  |  | N stage |  |  |  |
| N0 | 8 | 7/1 | 0.09 | N0 | 14 | 6/8 | 0.706 |
| N1-3 | 19 | 9/10 |  | N1-3 | 13 | 7/6 |  |
| Nx | 2 | 0/2 |  | Nx | 8 | 5/3 |  |
| NA | 2 | 0/2 |  | NA | 1 | 0/1 |  |
| M stage |  |  |  | M stage |  |  |  |
| M0 | 18 | 11/7 | 1.00 | M0 | 12 | 6/6 | 1.00 |
| M1 | 0 | 0/0 |  | M1 | 0 | 0/0 |  |
| Mx | 6 | 4/2 |  | Mx | 6 | 3/3 |  |
| NA | 7 | 1/6 |  | NA | 18 | 9/9 |  |

NA = Not Applicable.
