# Supplementary material for: The Expression and Effection of MicroRNA-499a in High-Tobacco Exposed Head and Neck Squamous Cell Carcinoma: A Bioinformatic Analysis
Source: Front Oncol. 2019 Jul 31;9:678. doi: 10.3389/fonc.2019.00678 (PMC6685408; doi:10.3389/fonc.2019.00678)
Supplement: Supplementary file 11 [file Table_11.docx]

**Supplementary Figure 1.** Survival curve for the three groups of HNSCC using Kaplan-Meier analyses (log-rank method). In low-tobacco (A, P = 0.39) and medium-tobacco (B, P = 0.66) exposed HNSCC, the patients with various expression levels of hsa-miR-129-2 were not different in overall survival. In high-tobacco exposed HNSCC, the patients with low expression of hsa-miR-129-2 had higher overall survival rates than the ones with high expression (C, P = 0.047).

**Supplementary Figure 2.** Survival curve for the three groups of HNSCC using Kaplan-Meier analyses (log-rank method). In low-tobacco (A, P = 0.28) and medium-tobacco (B, P = 0.72) exposed HNSCC, the patients with various expression levels of hsa-miR-508 were not different in overall survival. In high-tobacco exposed HNSCC, the patients with high expression of hsa-miR-508 had higher overall survival rates than the ones with low expression (C, P = 0.044).
