# Supplementary figures and images for: The Expression and Effection of MicroRNA-499a in High-Tobacco Exposed Head and Neck Squamous Cell Carcinoma: A Bioinformatic Analysis
Source: Front Oncol. 2019 Jul 31;9:678. doi: 10.3389/fonc.2019.00678 (PMC6685408; doi:10.3389/fonc.2019.00678)

## Supplementary Figure 1

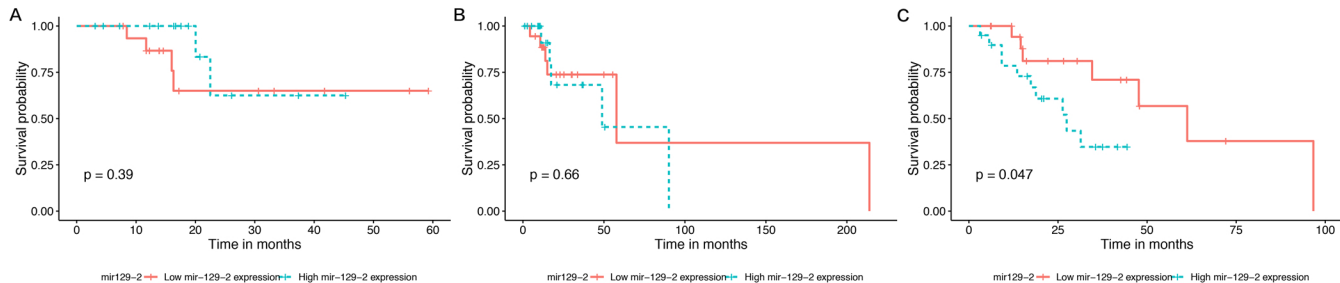

## Supplementary Figure 2

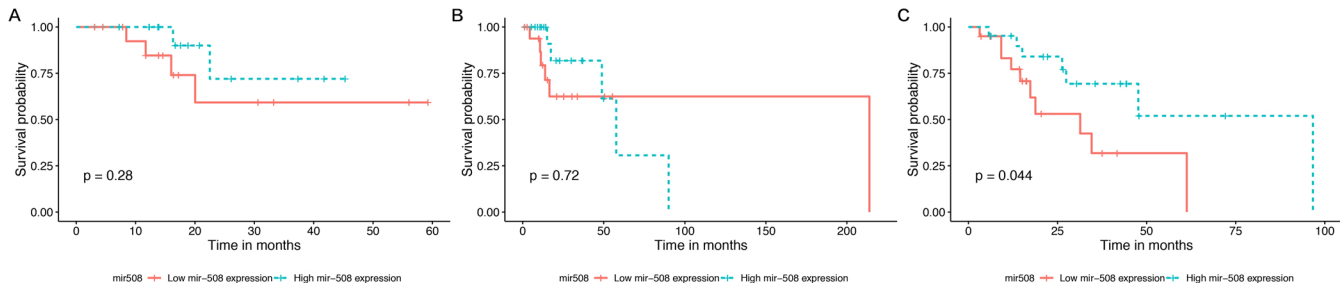

Supplement: Supplementary file 12 [file Data_Sheet_1.PDF]
